# Supplementary material for: Redox-controlled reorganization and flavin strain within the ribonucleotide reductase R2b–NrdI complex monitored by serial femtosecond crystallography
Source: eLife. 2022 Sep 9;11:e79226. doi: 10.7554/eLife.79226 (PMC9462851; doi:10.7554/eLife.79226)
Supplement: Supplementary file 1. — The contacts of FMN to NrdI are not listed since those are also formed in the absence of R2b. The only additional contacts formed with between FMN and R2b are with the side chains (sc) of Phe17 and Gln20. The contacts formed by those side chains are also given. Contacts in both the oxidized and reduced structures are listed. [file elife-79226-supp1.docx]

|  | ***Bc*R2b_MnMn_-NrdI_ox_** | | | ***Bc*R2b_MnMn_-NrdI_hq_** | | |
| --- | --- | --- | --- | --- | --- | --- |
|  | **FMN** | **R2b/Gln20 sc** | **R2b/Phe17 sc** | **FMN** | **R2b/Gln20 sc** | **R2b/Phe17 sc** |
| Hydrogen bonds | -- | R2b/Gln24 | -- | -- | R2b/Gln24 | -- |
| Non-polar contacts | R2b/Phe17 sc | R2b/Asn21 | R2b/Phe13 | R2b/Phe17 sc | R2b/Asn21 | R2b/Phe13 |
|  | R2b/Gln20 sc | R2b/Gln24 | R2b/Met16 | R2b/Glu20 sc | R2b/Gln24 | R2b/Met16 |
|  |  | R2b/Ile197 | R2b/Ile197 |  | R2b/Ile197 | R2b/Ile197 |
|  |  | NrdI/Gly44 | R2b/Val200 |  | NrdI/Gly44 | R2b/Val200 |
|  |  | NrdI/Phe45 | R2b/Phe201 |  | NrdI/Phe45 | R2b/Phe201 |
|  |  | NrdI/Trp74 | R2b/Ile204 |  |  |  |
