# Supplementary material for: Redox-controlled reorganization and flavin strain within the ribonucleotide reductase R2b–NrdI complex monitored by serial femtosecond crystallography
Source: eLife. 2022 Sep 9;11:e79226. doi: 10.7554/eLife.79226 (PMC9462851; doi:10.7554/eLife.79226)
Supplement: Supplementary file 2. — The strain energies are computed relative to the same redox state of the flavin, optimized without the protein surroundings. See Materials and methods for definition of the bending angle. [file elife-79226-supp2.docx]

|  | **NrdI** | | **R2b-NrdI** | | **(R2b-NrdI) – NrdI** |
| --- | --- | --- | --- | --- | --- |
|  | **Bending angle** | ***E*_strain_** (eV) | **Bending angle** | ***E*_strain_** (eV) | **Δ*E*_strain_** (meV) |
| FMN_ox_ | -2.0° | 0.61 | +1.8° | 0.76 | 151 |
| FMN_sq_ | +4.9° | 0.81 | -3.2° | 0.87 | 65 |
| FMN_hq_ | +11.1° | 0.73 | +4.1° | 0.80 | 75 |
