# Supplementary material for: Redox-controlled reorganization and flavin strain within the ribonucleotide reductase R2b–NrdI complex monitored by serial femtosecond crystallography
Source: eLife. 2022 Sep 9;11:e79226. doi: 10.7554/eLife.79226 (PMC9462851; doi:10.7554/eLife.79226)
Supplement: Supplementary file 3. — The relative redox potentials, ΔEtot, are computed at the B3LYP-D3/def2-TZVP/ε = 4 level relative to the same redox transition of the isolated flavin in ε = 4. The shifts in strain energy (at ε = 4), ΔEstrain, refer to the same redox transition relative to the isolated flavin, optimized without surroundings. The protein electrostatic shift, ΔEel, are estimated based on the difference between ΔEtot and ΔEstrain (ΔEtot = ΔEstrain+ ΔEel). ΔΔEtot are the total shifts in redox potentials of R2b–NrdI relative to NrdI alone. [file elife-79226-supp3.docx]

|  | **NrdI (mV)** (relative to isolated flavin) | | | **R2b-NrdI (mV)** (relative to isolated flavin) | | | **ΔΔ*E*_tot_ (mV)** (R2b-NrdI) – NrdI |
| --- | --- | --- | --- | --- | --- | --- | --- |
|  | Δ*E*_strain_ | Δ*E*_el_ | **Δ*E*_tot_** | Δ*E*_strain_ | Δ*E*_el_ | **Δ*E*_tot_** |  |
| FMN_sq_/FMN_ox_ | +129 | -397 | -268 | +86 | -235 | -150 | **118** |
| FMN_hq_/FMN_sq_ | -12 | +126 | +114 | -20 | +187 | +168 | **54** |
